# Patterns of infection, origins, and transmission of ranaviruses among the ectothermic vertebrates of Asia

**DOI:** 10.1002/ece3.8243

**Published:** 2021-10-25

**Authors:** Jayampathi Herath, Gajaba Ellepola, Madhava Meegaskumbura

**Affiliations:** ^1^ College of Forestry Guangxi Key Lab for Forest Ecology and Conservation Guangxi University Nanning China; ^2^ Department of Zoology Faculty of Science University of Peradeniya Kandy Sri Lanka

**Keywords:** animal trade, biosecurity, emerging diseases, ranavirosis, seasonality

## Abstract

Ranaviral infections, a malady of ectothermic vertebrates, are becoming frequent, severe, and widespread, causing mortality among both wild and cultured species, raising odds of species extinctions and economic losses. This increase in infection is possibly due to the broad host range of ranaviruses and the transmission of these pathogens through regional and international trade in Asia, where outbreaks have been increasingly reported over the past decade. Here, we focus attention on the origins, means of transmission, and patterns of spread of this infection within the region. Infections have been recorded in both cultured and wild populations in at least nine countries/administrative regions, together with mass die‐offs in some regions. Despite the imminent seriousness of the disease in Asia, surveillance efforts are still incipient. Some of the viral strains within Asia may transmit across host–taxon barriers, posing a significant risk to native species. Factors such as rising temperatures due to global climate change seem to exacerbate ranaviral activity, as most known outbreaks have been recorded during summer; however, data are still inadequate to verify this pattern for Asia. Import risk analysis, using protocols such as Pandora+, pre‐border pathogen screening, and effective biosecurity measures, can be used to mitigate introduction of ranaviruses to uninfected areas and curb transmission within Asia. Comprehensive surveillance using molecular diagnostic tools for ranavirus species and variants will help in understanding the prevalence and disease burden in the region. This is an important step toward conserving native biodiversity and safeguarding the aquaculture industry.

## Graphical abstract

Ranaviral infections are becoming frequent, severe, and widespread, causing mortality in native biodiversity and cultured species in Asia. Ranaviruses have a broad host range and are transmitted through live animal trade. Ranaviral disease is predominantly a summer disease, which may spread unhindered with global warming. The controlling of the disease in Asia will need effective biosecurity measures including use of protocols such as Pandora+ and pre‐border pathogen screening.

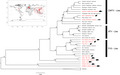

## 1 INTRODUCTION

Asia has been identified as an epicenter of emerging infectious diseases (EIDs) that display a complex relationship between host populations and the environment, involving domestic animals, wildlife, and human populations (Yadav et al., 2020). Ranaviruses are pathogenic agents capable of infecting all classes of ectothermic vertebrates, causing significant morbidity and mortality depending on the specific virus, host, and environmental factors (Gray & Chinchar, 2015). Given the broad host range and propensity to spread rapidly through regional and international trade, ranaviruses could contribute to species declines and pose a significant threat to cultured species and wildlife (Brunner et al., 2015).

Here, we first outline the general background of the disease in terms of host range, major outbreaks and severity, population declines and recovery, optimal climate, and diagnostic techniques. We then assess patterns of infection, origins, and transmission of ranavirus infections in Asia while concentrating specifically on the following: (1) We compile recorded ranavirus infections by country, highlighting clinical presentation in hosts, seasonality, incidences of mass die‐off events, whether hosts are wild or cultured species, native or alien, their likely origins, and the diagnostic tools used; (2) we evaluate potential routes of entry of ranavirus to Asia through cultured species; (3) we infer how climate change may affect ranavirus transmission dynamics in Asia; and (4) we present a set of recommendations for inhibiting further ranavirus transmissions to hitherto unaffected parts of Asia, and measures to strengthen biosecurity. We hope thereby to enhance understanding of ranavirus species, evaluate the disease burden, help quantifying risks, and identify host–pathogen interactions, epidemiology, and conservation concerns, in the context of the economic importance of the disease in Asia.

## 2 DISCOVERY OF RANAVIRUSES, HOST RANGE, AND OUTBREAKS

*Ranavirus* is one of the seven genera within the family Iridoviridae (International Committee on Taxonomy of Viruses, 2021a), a pathogen that infects ectothermic vertebrates such as bony fish, amphibians, and reptiles. The diseases resulting from infections by species of *Ranavirus* (hereafter, ranaviruses) are characterized by a short incubation period and high mortality (Allender, 2018), affecting the health of both free‐ranging and captive populations of hosts. Seven species of *Ranavirus* have been described according to the updated classification of the International Committee on Taxonomy of Viruses (ICTV): *Ambystoma tigrinum virus* (ATV), *Common midwife toad virus* (CMTV), *Epizootic hematopoietic necrosis virus* (EHNV), *European North Atlantic ranavirus* (LfRV), *Frog virus 3* (FV3), *Santee*‐*Cooper ranavirus*, and *Singapore grouper iridovirus* (SGIV). Ranavirus isolates are considered members of the same viral species if they share >95% amino acid identity (International Committee on Taxonomy of Viruses, 2021b). However, *Ranavirus maximus*, *Cod iridovirus*, and *Short*‐*finned eel virus* are potential new species that remain unclassified (International Committee on Taxonomy of Viruses, 2021b). The earlier classification, however, included *Frog virus 3* (FV3), *Ambystoma tigrinum virus* (ATV), *Bohle iridovirus* (BIV), *Epizootic hematopoietic necrosis virus* (EHNV*)*, *European catfish virus* (ECV), and *Santee*‐*Cooper ranavirus* (SCRV) (Jancovich et al., 2012). Among these, *Frog virus 3*, the type species of *Ranavirus*, was discovered originally in northern leopard frogs (*Lithobates pipiens*; Granoff et al., 1965) and ranaviruses are now known to infect at least 175 species across 52 families of ectothermic vertebrates on all continents except Antarctica (Duffus et al., 2015). The *Frog virus 3* group is restricted to amphibian hosts, whereas ATVs are predominantly fish specialists that made a single switch to caudate amphibians (Price et al., 2017). Further, “CMTV‐like” ranaviruses may circulate independently in amphibian and fish communities. By 2015, ranaviruses had been reported in at least 105 species of amphibians in 18 families across 25 countries (Duffus et al., 2015).

Most cases of ranavirosis have been recorded in North America, Europe, and Australia, with relatively few reported from Asia and Africa (Duffus et al., 2015). Outbreaks of FV3 and FV3‐like viruses in amphibians have been detected in many different species of anurans and urodeles around the world: 42 amphibians from 9 species (an overall prevalence of 16.6%) tested positive for ranaviruses in Costa Rica (Whitfield et al., 2013); FV3‐like ranaviruses in five amphibian species in Europe (Gray & Chinchar, 2015); and FV3 infection in wild three‐spine stickleback fish in the eastern continental USA (Mao et al., 1999). Despite the seeming host specificity outlined here, ranaviruses can cross taxon barriers.

The ability of ranaviruses to cross species barriers can give rise to catastrophic consequences (Price et al., 2014), especially in small populations where recovery is slow (Earl & Gray, 2014; Price et al., 2014). Ranavirus die‐off sites indicate up to 80% declines in common frog abundance in England (Teacher et al., 2010). In addition, at sites where ranavirus die‐offs occurred, amphibian recruitment attenuated in consecutive years (Petranka et al., 2003), signifying poor recovery following population declines. Another intriguing pattern in ranavirosis is the variation in mortality ranging from absence to massive die‐offs (Gray et al., 2009), where stressors can play an important role.

## 3 ANTHROPOGENIC EFFECTS

Environmental and anthropogenic stressors are important in disease outbreaks leading to mass mortality, with the latter playing a dominant role. For instance, the prevalence of ranaviral infection in green frog (*Lithobates clamitans*) populations increases with proximity to industry and housing, but the underlying mechanisms are unclear (Daszak et al., 2001; St‐Amour et al., 2008). Immunosuppressive corticosterone (CORT), wetland water quality deterioration due to cattle, water pollutants from unconventional oil and gas extraction (UOG), and salinity‐related stress may also reduce tolerance to infection, thus increasing mortality in amphibians (Davis et al., 2019; Gray et al., 2007; Hall et al., 2020; Robert et al., 2019).

## 4 SEASONALITY

Another factor influencing ranavirosis is seasonality. The present understanding of the seasonality of ranavirus diseases derives mostly from studies outside of Asia. In amphibians and fish, there is usually a rapid onset of ranaviral epidemics in the mid‐to‐late Northern Hemisphere summer, while outbreaks among reptiles seem to be irregular. Two distinct patterns of die‐offs in farmed and wild populations of amphibians, fish, and reptiles attributed to ranavirus have been observed (Brunner et al., 2015). The first shows rapid onset of die‐offs, usually occurring in fish and amphibians during summer months. The second shows variable vulnerability of host populations, as mentioned earlier, ranging from absence of mortality to near‐complete die‐offs.

An environmental DNA‐based study in Wood frogs (*L*. *sylvatica*) found that the timing of pathogen introduction did not affect the timing of epidemics or the resulting die‐offs, and instead, timing appears to be driven by development and/or temperature‐dependent changes in pathogenicity (Hall et al., 2018). Outbreaks of EHNV infecting redfin perch (*Perca fluviatilis*) in northeastern Victoria, Australia, were recorded in November–December (early summer in Southern Hemisphere) (Langdon et al., 1986). Incidence of LMBV in Largemouth bass die‐offs was associated with increased summer temperatures (Grizzle & Brunner, 2003). However, when ranavirus epidemics occur during late spring or summer, they are of shorter duration—only lasting for a few weeks (Green et al., 2002). Broadly, these studies indicate greater disease incidence during the summer months.

Only a few studies report seasonality in ranavirus outbreaks in Asia. However, the few outbreaks recorded so far involved Asian amphibians during summer, mostly in cultured species when mass mortality occurred. These include the following: in exotic North American bullfrog (*Lithobates catesbeianus*, formerly *Rana catesbeiana*) larvae in Japan from early September to mid‐October (Une et al., 2009); in cultured Chinese giant salamanders (*Andrias davidianus*) between February and November (Chen et al., 2013; Cunningham et al., 2016; Du et al., 2016; Geng et al., 2011); in black‐spotted pond frogs (*Rana nigromaculata*) in March; and in cultured Chinese tiger frog (*Hoplobatrachus* cf. *rugulosus*, formerly *R*. *tigrina rugulosa)* between May and June (Mu et al., 2018; Weng et al., 2002). However, generalizing seasonality of ranavirosis based on a few studies is inadequate as there may be significant geographic variation and some of these patterns may already be altering due to climatic change.

## 5 RANAVIRUS INFECTIONS AND CLIMATIC CHANGE

Since temperature can influence both the kinetics of host–parasite interactions and act as a host stressor (Altizer et al., 2013), climate change may already be playing a significant role in ranavirosis. It has been noted that most die‐off events caused by ranaviruses begin (and often end) during the summer months (Brunner et al., 2015) and that various ranaviral strains respond differently to temperature. Under laboratory conditions, Short‐finned eel ranavirus (SERV) replicated optimally at 20°C (Ariel & Jensen, 2009), while LMBV grew slightly faster at 30°C than at 25°C (Grant et al., 2003). Higher mortality rates due to ranavirosis are in many cases associated with higher temperatures. For example, Redfin perch and rainbow trout infected with EHNV (Ariel & Jensen, 2009) and common frog tadpoles exposed to ranaviruses (Gray et al., 2009) showed increased mortality with higher temperatures. Although there are a few exceptions (Allender et al., 2018; Rojas et al., 2005), most studies suggest that higher temperatures stress host species allowing ranavirus infections to proliferate (Ariel & Jensen, 2009; Bayley et al., 2013; Echaubard et al., 2014; Price et al., 2019).

Climate projections suggest that global warming will likely play a significant role in shaping future ranavirus disease dynamics in amphibians, altering both geographic extent and length of temporal window for disease risk and severity (Price et al., 2019). Furthermore, unprecedented urbanization in Asia, together with associated temperature increases (Song et al., 2003; Wang, Zhang, et al., 2014; Wang, Ji, et al., 2014), may exacerbate ranaviral outbreaks in the region.

## 6 DIAGNOSTIC TESTS

Earlier methods for detection of ranaviruses included electron microscopy (EM), histopathology, and cytology, and newer approaches include virus isolation, antigen‐capture enzyme‐linked immunosorbent assay (Ag‐capture ELISA), immunohistochemistry (IHC), and PCR (Miller et al., 2015). In both conventional and real‐time PCR (qPCR), the major capsid protein (MCP) gene, neurofilament triplet H1 protein, DNA polymerase, and an intergenic variable region can be used as targets (Holopainen et al., 2009; Hyatt et al., 2000; Jancovich et al., 2005; Mao et al., 1997). PCR is currently the commonly used approach for ranavirus detection.

## 7 RANAVIRUS INFECTIONS IN ASIA

**FIGURE 1:**
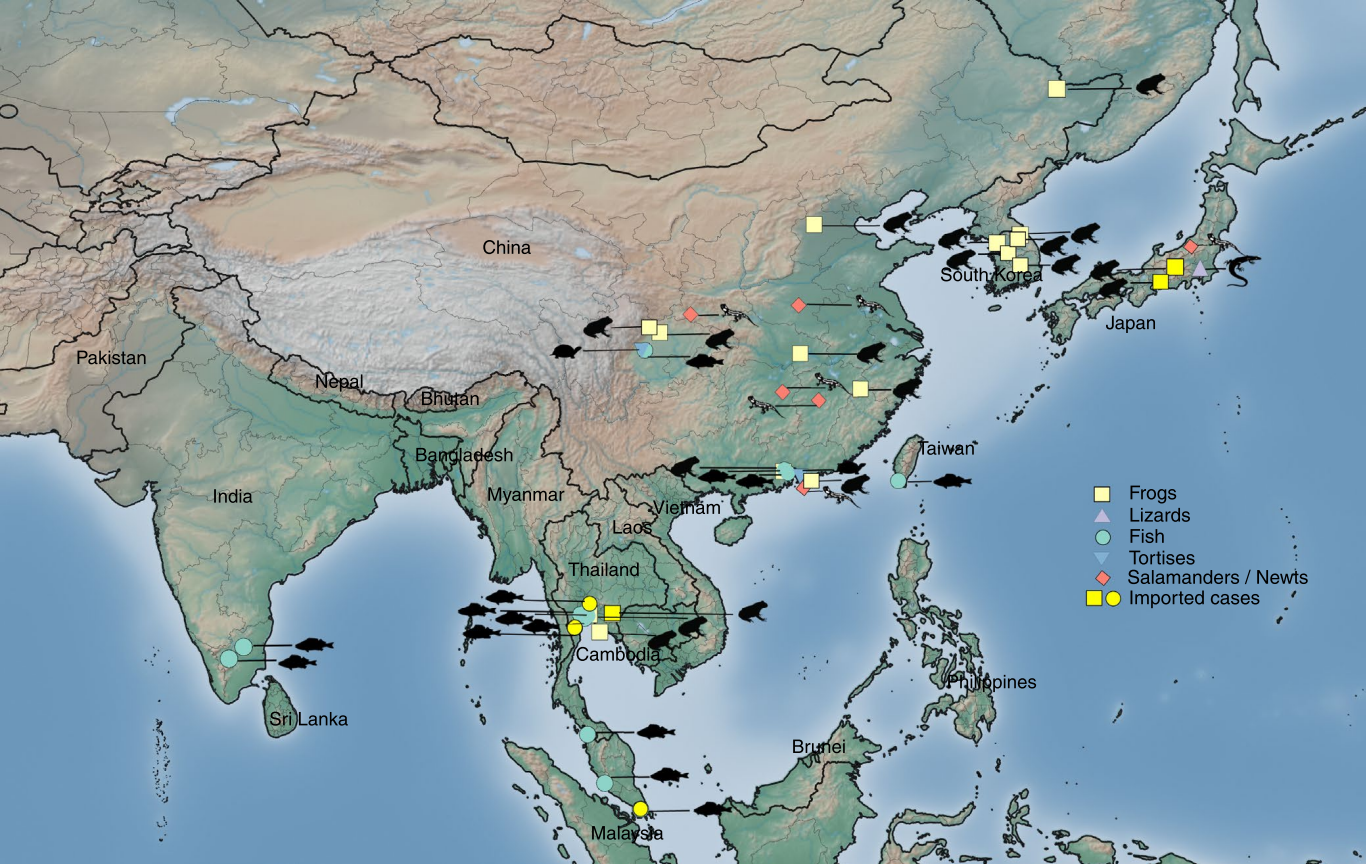
Regional distribution of Asian ranavirus cases by country. Hosts of ranaviruses (salamanders/newts, anurans, fish, agamid lizards, and testudines) are indicated by the respective silhouettes of hosts

Ranaviruses have been recorded in several countries in the region. These include China, Taiwan, Hong Kong, Japan, South Korea, Thailand, Singapore, Malaysia, and India (Chen et al., 1999, 2013; Cunningham et al., 2016; Deng et al., 2011, 2020; Du et al., 2016; Fu et al., 2017; Geng et al., 2011; George et al., 2015; Hazeri et al., 2016, 2017; He et al., 2002; Huang et al., 2009; Kayansamruaj et al., 2017; Kim et al., 2009; Kolby et al., 2014; Kwon et al., 2017; Lai et al., 2000; Mu et al., 2018; Murali et al., 2002; Park et al., 2017, 2021; Qin et al., 2003; Sivasankar et al., 2017; Sriwanayos et al., 2020; Tamukai et al., 2016; Une et al., 2009, 2014; Une, Sakuma, et al., 2009; Weng et al., 2002; Xu et al., 2010; Yu et al., 2015, 2020; Yuan et al., 2016; Zhang et al., 2001; Zhu & Wang, 2016) (Figure 1).

**FIGURE 2:**
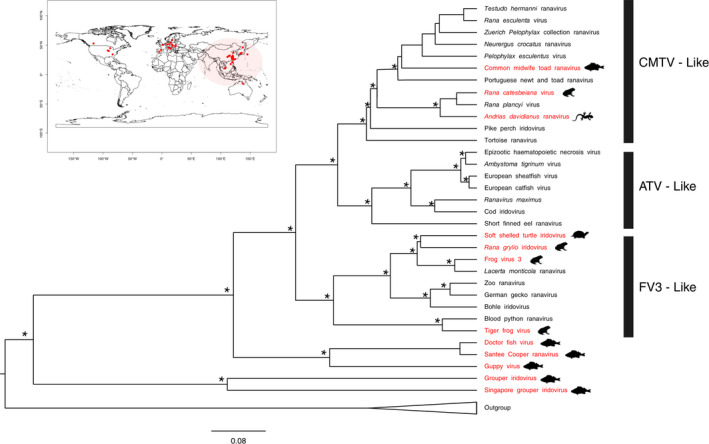
Asian ranaviruses on a phylogeny: Phylogenetic perspective of Asian ranaviruses (highlighted in red) in the context of broad virus type (Frog virus (FV3)‐like, Common midwife toad virus (CMTV)‐like, and Ambystoma tigrinum virus (ATV)‐like). Hosts of Asian ranaviruses (salamanders/newts, anurans, fish, and testudines) are indicated by the respective silhouettes of hosts. The overall topology of the tree was obtained by a two‐stage Bayesian approach PASTIS (Thomas et al., 2013), which uses a backbone topology based on molecular data, a set of taxonomic postulates and user‐defined priors on branch lengths and topologies to generate a posterior distribution of complete ultrametric trees that capture uncertainty under a homogeneous birth–death prior model of diversification and placement constraints. Twenty‐five complete genomes (~100,000 bp) deposited in GenBank (last accessed on February 2021) were aligned in MAFFT and were analyzed in several tree‐building programs such as MAFFT, IQtree, NDtree, and CSI phylogeny, using the best‐fitting model as TVMe+ R2, which provided topologies congruent with those of Chinchar et al. (2017). The tree output given by the MAFFT (i.e., the ultrametric tree) was used as the backbone tree in the analysis. To incorporate isolates with short sequence data into the backbone tree, molecular data for several loci (MCP, DPG, RDRASPG, and RDRBS) for all taxa included in the tree were downloaded from GenBank. They were aligned in MEGA using MUSCLE, were softly constrained into their specific broad virus type based on the taxonomic information available in the literature using PASTIS, and were analyzed in MrBayes by running for 60 million generations. As soft constraints were allowed to move freely on branches, they were able to fix at places with maximum‐likelihood values, revealing possible relationships of taxa whose phylogenetic positions were previously unknown or doubtful. Branches with posterior probability values >95% are marked by asterisks. Lymphocystis disease virus from China was used as the outgroup. The world map in the subfigure shows known locations of ranaviruses, in which the highlighted extent depicts the Asian region considered in this paper. Note: For detailed descriptions of ranavirus isolates in Asia, see Table 1. Inland bearded dragon ranavirus, Koi ranavirus, Oxyeleotris ranavirus, Goldfish ranavirus, Asian grass frog ranavirus, and East Asian bull frog ranavirus are not indicated in the phylogeny as their phylogenetic relationships were unspecified or doubtful

The first case of ranavirus infection in Asia was reported in the cultured pig frog (*Lithobates grylio*, formerly *Rana grylio*) in China (Zhang et al., 1996, 2001). Only a few countries carry out surveillance, and the preponderance of monitoring has been confined to species that are cultured either for food or for the pet industry. The limited intensity of surveillance implies a high probability of ranaviruses infecting wild species undetected (Kwon et al., 2017; Park et al., 2021; Zhu & Wang, 2016). So far, the region harbors four out of seven ranavirus species (or ranavirus isolates considered members of the same viral species) recognized by the International Committee on Taxonomy of Viruses (Figure 2). Ambystoma tigrinum virus, Epizootic hematopoietic necrosis virus, and European North Atlantic ranavirus (or their isolates) are the only three species that have not yet been reported from the region.

### 7.1 Mainland China

Ranavirus infections have been recorded in all ectothermic vertebrate groups in China. The screening efforts remain modest considering the risks of ranaviral infection due to intensive aquaculture, ranaculture, and mariculture, as well as the pet industry (Figure 1).

Ranaviruses have caused dramatic declines of the wild populations of Chinese giant salamander (*A*. *davidianus*), a caudate and a species of conservation importance. The first report of ranavirus‐driven mass mortality in Chinese giant salamanders (*A*. *davidianus*) was recorded among farmed populations in Shanxi Province (Geng et al., 2011), where all specimens collected tested positive for ranavirus. The Chinese giant salamander is one of the largest amphibians in the world, and it is Critically Endangered (IUCN Red List, 2012) because of threats such as habitat loss and overharvesting. Emerging infectious diseases such as the Chinese giant salamander iridovirus (CGSIV) can also cause population declines, with mass mortality events increasing their extinction risk (Dong et al., 2011; Meng et al., 2014; Wang, Zhang, et al., 2014; Wang, Ji, et al., 2014). Adult Chinese giant salamanders have been shown to be infected in natural habitats and/or farms in Hunan, Jiangxi, and Henan Provinces during 2011–2012 (Chen et al., 2013). The isolate responsible for this infection was provisionally designated as Andrias davidianus ranavirus (ADRV).

In terms of species, anurans are the group that is mostly infected with ranavirus in China. A study of wild Dybowski's frogs (*Rana dybowskii*) in Heilongjiang Province showed that the overall infection prevalence is 5.7% in adults and 42.5% in tadpoles (Xu et al., 2010). Overall prevalence of infection in adults was shown to be low compared with tadpoles; neither showed clinical signs. Another study highlighted ten strains of FV3‐like ranavirus in wild, native *R*. *dybowskii* and *R*. *amurensis* in northeastern China (Zhu & Wang, 2016). Eight of these strains were isolated from *R*. *dybowskii* from Heihe, Hebei, and Dongfanghong, while the remaining two were detected in *R*. *amurensis* from Hebei. These 10 strains were homologous to FV3, though genetic differences were noted among the isolates. “Frog Virus 3 (FV3)”‐like iridoviruses can cause widespread, severe disease and mortality among cultured pig frogs (*L*. *grylio*), and farms in Hubei Province and Hunan Province have experienced mortality rates above 90% (Zhang et al., 2001). Another outbreak was recorded in cultured Chinese edible frogs (*Hoplobatrachus rugulosus*) in culture facilities in Guangdong and Hainan Provinces, which was also closely related to FV3 (Weng et al., 2002). Tiger frog virus in a captive population of *H*. *rugulosus* in Nanhai, Guangdong, in Southern China, caused high mortality of tadpoles (He et al., 2002; Yuan et al., 2016). Further, tadpoles of a cultured population of black‐spotted pond frogs (*R*. *nigromaculata*) in Shuangliu County, China, were diagnosed with a FV3‐like ranavirus infection where substantial mortality was reported (90%; Mu et al., 2018). Similar outbreak among black‐spotted pond frogs (*R*. *nigromaculata*) was recorded in tadpoles from a frog farm located in Shuangliu (Sichuan Province) in 2017 (Yu et al., 2020).

Fishes of economic value have been affected by ranaviruses. High mortality in cultured largemouth bass (*Micropterus salmoides*) was observed in Foshan, Guangdong Province, in 2008, and the virus was identified as identical to doctor fish virus (DFV), a pathogen closely related to largemouth bass virus (LMBV) (Deng et al., 2011). Another high mortality event occurred due to “Common midwife toad virus (CMTV)”‐like ranavirus in a cultured loach (*Triplophysa siluorides*) in Sichuan Province, which affected about 75% of the stock (Deng et al., 2020). Isolates of Santee‐Cooper ranaviruses from diseased Chinese perch (*Siniperca chuatsi*) and snakehead fish (*Channa maculata*) from Guangdong Province suggest that infection may be widespread in China (Fu et al., 2017).

In contrast to fishes, only two species of reptile have been found to be infected so far. Soft‐shelled turtle iridovirus (STIV) causing “red neck disease” in farmed soft‐shelled turtle (*Trionyx sinensis*) has been recorded in Shenzhen (Chen et al., 1999). Phylogenetic analyses suggested that STIV and FV3 are closely related and may transmit between reptiles and amphibians (Huang et al., 2009). A ranavirus in testudines was recorded in snapping turtles (*Macrochelys temminckii*) in Sichuan Province (Yu et al., 2015).

### 7.2 Hong Kong SAR, China

Ranaviruses have been detected in 105 of 185 (56.8%) of the number of individual amphibians tested, which were exported from Hong Kong to the USA (Kolby et al., 2014). Ranaviruses were identified in oriental fire‐bellied toad (*Bombina orientalis*), oriental fire‐bellied newt (*Cynops orientalis*), and Hong Kong newt (*Paramesotriton hongkongensis*). However, it is not known whether the infection is only found among traded species or whether native amphibians in Hong Kong are already infected.

### 7.3 Taiwan, China

Only fish have been recorded with ranaviruses so far in Taiwan. Grouper iridovirus (GIV) was isolated from yellow groupers (*Epinephelus awoara*) in a fish farm in Hsiau Liouchiou Island (Lai et al., 2000; Murali et al., 2002), where mortality reached 100% within 11 to 25 days post‐infection (Murali et al., 2002). Furthermore, twenty‐three iridovirus isolates from both seawater and freshwater fish from farms distributed throughout all seven Taiwan prefectures have been recorded between 2001 and 2009 (Huang et al., 2011). These isolates were collected from eight species of cultured fish: 11 isolates from giant grouper (*Epinephelus lanceolatus*), five from orange‐spotted grouper (*E*. *coioides*), three from giant seaperch (*Lates calcarifer*), one from crimson snapper (*Lutjanus erythropterus*), one from silver sea bream (*Rhabdosargus sarba*), one from largemouth bass (*M*. *salmoides*), one from rock bream (*Oplegnathus fasciatus*), and one from marble goby (*Oxyeleotris marmoratus*). The 23 isolates were divided into six groups within the genera *Ranavirus* and *Megalocytivirus*. (Huang et al., 2011). The phylogenetic analysis of viral genomic DNA based on the MCP genes showed that the genotypes of these isolates were closely related to SGIV and GIV. Given the wide range of habitats and high number of potential host species (76 reptiles, 30 amphibians, and 130 freshwater fishes) present in Taiwan (WWF, 2020), the propensity for the pathogen to spread should be closely monitored.

### 7.4 Japan

Biogeographically isolated from the rest of Asia by the Sea of Japan, Japan harbors a high amphibian diversity. These amphibian species have a high risk of acquiring ranaviruses from imported aquatic organisms. Wild North American bullfrog (*L*. *catesbeiana*) larvae suffered a mass die‐off in a pond in western Japan during the autumn of 2008, attributed to RCV‐JP (Une, Sakuma, et al., 2009). Another outbreak was reported in a captive collection, which involved several species of poison dart frogs (*Dendrobates* spp. and *Phyllobates* spp.); this infection was triggered following an introduction of imported *Dendrobates* spp. from the Netherlands in 2012 (Une et al., 2014). An outbreak occurred in a captive colony of Japanese clouded salamander (*Hynobius nebulosus*), and the entire colony was destroyed in 20 days (Une, Nakajima, et al., 2009).

The most recent reported ranavirus outbreak was detected in a population of inland bearded dragons (*Pogona vitticeps*; *n* = 100) at a breeding facility in Japan. This was named as the inland bearded dragon ranavirus (IBDRV), and MCP gene sequence analysis showed it to be similar to the three ranaviruses described in infected amphibians in Japan, Korea, and Taiwan. Reptilian ranaviruses, which often cluster closely with amphibian ranaviruses (“FV3‐like,” “TFV‐like,” or “CMTV‐like”), were also detected in the vicinity of the breeding facility, from which horizontal transmission may have occurred (Tamukai et al., 2016).

### 7.5 South Korea

There are five cases of ranaviral infections reported from South Korea. The first of these was a mass mortality event in a natural population of huanren frog (*R*. *huanrenensis*) tadpoles by a ranavirus closely related to the Rana catesbeiana virus JP MCP, isolated from invasive bullfrog tadpoles in Japan (Kwon et al., 2017). This is perhaps the best‐known case of ranavirosis implicated in a mass mortality event of an endemic wild amphibian in Asia. Another mass mortality event involving an adult population of Dybowski's brown frogs (*R*. *dybowskii*) was detected in 2017, from a stream Moksang‐dong (Park et al., 2021). The MCP sequence resembled the Frog virus 3 (FV3) that had been collected earlier from huanren brown frog (*R*. *huanrenensis*) tadpoles in South Korea. Dead adults of boreal digging frog (*Kaloula borealis*) from Eoeun‐dong, Daejeon‐si, were confirmed to be infected with ranavirus, while tadpoles of the Japanese tree frog (*Hyla japonica*) from Ugok‐ri, Yongju‐myeon, were also confirmed to be infected (Park et al., 2017). Last case from South Korea was reported from gold‐spotted pond frogs (*Pelophylax chosenicus*, formerly *Rana plancyi chosenica*) (Kim et al., 2009). Both the tadpoles and adults were infected in a culture facility, and the virus resembled Frog Virus 3 (FV3).

### 7.6 Thailand

Several ranaviruses were isolated from Thailand's aquaculture facilities between 1998 and 2001 (Sriwanayos et al., 2020). These were identified in marbled sleeper goby (*Oxyeleotris marmorata*), goldfish (*Carassius auratus*), guppy (*Poecilia reticulata*), tiger frog (*H*. *tigerinus*), Asian grass frog (*Fejervarya limnocharis*), and East Asian bullfrog (*H*. *rugulosus*). Asian grass frogs (*F*. *limnocharis)* had been imported from Cambodia, while the other species were cultured in situ. Phylogenomic analyses implicated eight Thai ranaviruses, which showed similarity to Chinese Tiger Frog Virus (TFV) and Wamena virus (WV), as a subclade within a larger frog virus 3 clade. Further, feeding of juvenile frogs to large predatory ornamental fishes may also have contributed to the spread of TFVs in Thailand. Another ranavirus infection associated with an outbreak of ulcerative disease among barcoo grunter fish (*Scortum barcoo*) in farms in the central region of Thailand (Ayutthaya and Phetchaburi provinces) was detected in 2013 (Kayansamruaj et al., 2017). These freshwater fish, a popular aquaculture species, are native to Australia and had recently been introduced to Thailand.

### 7.7 Singapore

Groupers are the only group detected with ranaviruses in Singapore. A newly isolated grouper virus from a diseased brown‐spotted grouper (*Epinephelus tauvina*), related to largemouth bass virus (LMBV), FV3, and Regina ranavirus (RRV), was named as Singapore grouper iridovirus (SGIV; Qin et al., 2003). SGIV was shown to cause serious systemic disease capable of killing 96% of grouper fry. Mariculture of *Epinephelus* species is rapidly developing in Singapore and other Southeast Asian countries, to which this virus poses a serious threat. Outbreaks of a novel viral disease called “sleepy grouper disease” (SGD) was first observed in *E*. *tauvina* in Singapore in 1994 (electron microscopic analyses), causing economic losses (Chua et al., 1994). However, the virus strain was not verified at the time by cell culture techniques. Later, it was identified as SGIV. Another outbreak of the same disease occurred in fry and adults of brown‐spotted groupers in 1998. These fry were imported from other Southeast Asian countries, and the outbreak lasted several weeks, resulting in more than 90% mortality.

### 7.8 India

There are two instances of ranavirus infections recorded from India. The first was in freshwater fishes, following a mass mortality event. A virus resembling Santee‐Cooper ranavirus was detected from koi carp (*Cyprinus rubrofuscus*) in ornamental fish farms of South India (George et al., 2015). The second case was reported from farm‐reared similar damselfish (*Pomacentrus similis*) with frequent mortality events reported from marine ornamental fish farms of South India (Sivasankar et al., 2017). The name “Similar damselfish virus” (SRDV) was proposed, and the MCP gene showed a close relationship to largemouth bass virus (LMBV).

### 7.9 Cambodia and Vietnam

The only study conducted so far in Cambodia and Vietnam failed to detect ranavirus in these countries (Gilbert et al., 2012). The screening was based on qPCR and histopathology of liver and other tissue samples collected from 74 frogs, with most samples being from Cambodia (n = 70).

### 7.10 The Philippines

A study carried out to investigate ranaviruses along with *Batrachochytrium dendrobatidis* among wild amphibians (*n* = 304, from seven sites) from the Philippine islands of Luzon, Negros, Calayan, and Camiguin Norte failed to detect ranaviruses (Smith et al., 2019).

### 7.11 Malaysia

Groupers are the only group detected with ranavirusses in Malaysia. Grouper iridovirus (GIV), phylogenetically closely related to other grouper iridoviruses from Asia, was recorded in both tiger grouper hybrid (*Epinephelus* sp.) and coral trout (*Plectropomus leopardus*; Hazeri et al., 2016; Hazeri et al., 2017).

## 8 SEVERITY OF RANAVIRUS INFECTIONS AT GLOBAL SCALE

Some host species are highly susceptible to ranaviruses, and experimental studies suggest that novel strains of ranaviruses introduced into native populations could have devastating consequences (Duffus et al., 2015). Despite limited monitoring efforts, the alarming increase in recent reports of ranavirus emergence in Asia may be an underlying reason for unexplained population declines such as in the case of Chinese giant salamanders (Dong et al., 2011; Meng et al., 2014; Wang, Zhang, et al., 2014; Wang, Ji, et al., 2014). High mortality rates of hosts and a diverse host range (and hence the potential to affect numerous novel species) have prompted the World Organization for Animal Health (OIE) to list ranavirus as a notifiable disease (i.e., transmissible diseases that have the potential for profoundly serious and rapid spread, irrespective of national borders, that entail serious socio‐economic or public health consequence and that are of major importance in the international trade of animals and animal products). Epizootic hematopoietic necrosis virus (EHNV) is listed as a fish disease, while infection by ranavirus species is listed as amphibian disease in the OIE’s listed diseases (World Organization for Animal Health, 2021). This designation requires countries (which have subscribed to OIE policies) to screen a sample of ranavirus hosts that cross international borders for ranaviruses (Schloegel et al., 2010). Quarterly Aquatic Animal Disease Report (Asia‐Pacific Region) includes data on disease prevalence of ranaviruses because the disease has become prevalent in the region (Network of Aquaculture Centers in Asia‐Pacific and Food, World Organization for Animal Health (OIE) Regional Representation for Asia, and the Pacific and Agriculture Organization of the United Nations, 2020),

## 9 INTRODUCING RANAVIRUSES TO UNINFECTED AREAS AND TRANSMISSION WITHIN ASIA

Growing international trade of live amphibians, reptiles, and fish, taken from the wild or bred in captivity, and sold commercially as food or ornamental species/pets, appears to increase the risk of introducing and dispersing ranaviruses across Asia. Several studies suggest that many species imported to Asia could potentially host ranaviruses (Table 1).

**TABLE 1:** Regional distribution of Asian ranavirus infections or mortality in wild and captive ectothermic vertebrates

Viral name (abbreviation)/phylogenetic relationship	Host species/life stage	Region/country	Wild/captive/introduced	Month/year	Suspected origin	Symptoms	Intensity	Detection method/phylogenetic analysis	Reference
Rana nigromaculata ranavirus (RNRV) (FV3‐like)	Black‐spotted pond frogs (*Rana nigromaculata*)/tadpoles	Shuangliu County, China	Cultured	April 2016	Breeding frogs bought from Guangdong Province of China. PCR indicates that the samples of breeding frogs and eggs were negative. But the water samples were positive. Potentially introduced from contaminated river water.	Hemorrhage on their body surface, swollen abdomen with yellow ascites, congestion, and swelling of the liver	High mortality (approximately 90% in tadpoles)	Virus isolation, electron microscopy, challenge experiments, PCR, MCP gene sequencing, and phylogenetic analysis	Mu et al. (2018)
Rana nigromaculata ranavirus (RNRV) (FV3‐like)	Black‐spotted pond frogs *(Rana nigromaculata)*/tadpoles	Shuangliu (Sichuan Province), China	Cultured	April to May 2017	Not available	Anorexia and swollen abdomen	High mortality	Electron microscopy, PCR, MCP gene sequencing, and phylogenetic analysis	Yu et al. (2020)
*Not specified 10 strains of FV3‐like ranavirus	*Rana dybowskii* *Rana amurensis*	Heihe, Hebei, and Dongfanghong, China	Wild	Not available	Potentially from introduced North American bullfrogs	Not available	Not available	Viral isolation, cloning, and sequencing	Zhu and Wang (2016)
*Not specified 98% homology with iridovirus RGV	*Rana dybowskii*	Hebei, Dongfanghong, Heihe, Tieli, Huanan, and Hailin regions, China	Wild	Not available	Not available	Not available	Moderate prevalence in tadpoles (42.5%) and low prevalence (5.7%) in adults	PCR, MCP gene sequencing, and phylogenetic analysis	Xu et al. (2010)
Tiger frog virus (TFV)	Tiger frog *(Hoplobatrachus tigerinus*, formerly *Rana tigrina rugulosa)* ‐ Tadpoles	Guangdong, China	Cultured	May to June2000	Not available	Abdominal distension, ataxia, reduced feeding	High mortality in tadpoles	Viral isolation, cloning and sequencing, Computer‐assisted analyses of the deduced amino acid sequences	Weng et al. (2002), He et al. (2002), Yuan et al. (2016)
Rana grylio virus (RGV) (Similar to FV3)	Pig frog (*Lithobates grylio*, formerly, *Rana grylio)*/adults	Wuhan, Hubei province, China	Cultured/introduced from the USA	1995–1998	Since *Rana grylio* is present in the southeast USA and has been imported into China for farming purposes, it is possible that imported frogs harbored the virus and served as the focus for the initial infection	Hemorrhages throughout the leg area. In young frogs, hemorrhagic spots around neck, back, and abdomen, and the skin began to ulcerate	High mortality (approximately 95%)	Virus isolation, electron microscopy, PCR, and nucleic acid sequence analysis	Zhang et al. (2001)
Chinese giant salamander virus (CGSV)—Provisional	Chinese Giant Salamanders (*Andrias Davidianus*)/adults	Hanzhong County, Shanxi Province, China	Cultured	February to May 2010	Not available	Anorexia, lethargy, ecchymoses, swollen areas on the head and limbs, and skin ulceration	High mortality	Virus isolation, electron microscopy, PCR, MCP gene sequencing, and phylogenetic analysis	Geng et al. (2011)
Chinese giant salamander (Andrias davidianus) iridovirus (CGSIV)	Chinese Giant Salamander (*Andrias davidianus*)/adults	Hanzhong County (Shaanxi Province, China)	Cultured	November 2014	Not available	shedding skin	Not available	Histological analysis, immunohistochemistry (IHC), immunofluorescence (IF), and RT‐PCR	Du et al. (2016)
Andrias davidianus ranavirus (ADRV) More closely related to frog ranaviruses than to other salamander ranaviruses	Chinese Giant Salamander (*Andrias davidianus*)/adults, larvae	Hunan, Jiangxi, and Henan Provinces of China	Cultured	May 2011 to August 2012	Not available	Severely hemorrhagic lesions	High mortality	Virus isolation, electron microscopy, challenge experiments, and genome annotation and analysis	Chen et al. (2013)
*Not specified (similar to Chinese giant salamander iridovirus and Rana grylio virus)	Chinese giant salamander (*Andrias davidianu*s)/adults	Shaanxi Province, China	Cultured	May 2010 to October 2011	Possibly by feeding salamanders with infected pig frogs	Swelling and bleeding of the head (known locally as big head disease) or feet (big foot disease), necrosis, and bleeding of the oral mucosa (bad mouth disease) or tail (bad tail disease), and skin bleeding.	Not available	PCR	Cunningham et al. (2016)
Santee‐Cooper ranavirus	Chinese perch (*Siniperca chuatsi*) and snakehead fish (*Channa maculate)*	Guangdong Province, China	Cultured	2015	Not available	Ascites, mesentery hemorrhages, and pus in the intestine. No specific external signs.	High mortality (100% mortality in challenge experiment) in *Siniperca chuatsi*	Virus isolation, electron microscopy, challenge experiments, PCR, MCP gene sequencing, and phylogenetic analysis	Fu et al. (2017)
*Not specified (may be identical to doctor fish virus (DFV) or a strain of DFV)	largemouth bass (*Micropterus salmoides*)	Foshan area of Guangdong Province, China	Cultured and introduced from the USA	June to October 2008	Potentially originated in USA	Ulcerations on the skin and muscle	High mortality (100% by challenge experiments)	Virus isolation, electron microscopy, challenge experiments, PCR, MCP gene sequencing, and phylogenetic analysis	Deng et al. (2011)
Triplophysa siluroides ranavirus (related to Common midwife toad virus (CMTV)‐like ranavirus clade)	Catfish‐like loach (*Triplophysa siluorides)*	Sichuan Province, China	Cultured	Not available	A Chinese giant salamander farm (with cases of CGSV infection) was located approximately 1 km upstream of the *T*. *siluroides* farm. Suspected to be transmitted by contaminated water from that farm	Skin lesions and hemorrhagic ulcers	Moderate‐to‐high mortality (40% to 90% by challenge experiments)	Virus isolation, electron microscopy, challenge experiments, MCP gene sequencing, and phylogenetic analysis	Deng et al. (2020)
Soft‐shelled turtle iridovirus (STIV)	Soft‐shelled turtle (*Trionyx sinensis*)	Shenzhen, China	Cultured	January 1997	Not available	Neck swelling and hemorrhage of the hypodermic area “Referred to as red neck disease”	Moderate mortality (less than 40% by challenge experiments)	Virus isolation, electron microscopy, and challenge experiments	Chen et al. (1999) Huang et al. (2009)
*Not specified	Alligator snapping turtles (*Macrochelys temminckii)*	Chengdu, Sichuan Province, China	Cultured/pet introduced	March 2013	Not available	Crawled in weakness, slowed response to external stimulation, local redness, and swelling in the neck and limbs	Not available	PCR, MCP gene sequencing, and phylogenetic analysis	Yu et al. (2015)
*Not specified	Fire‐bellied toad (*Bombina orientalis*), oriental fire‐bellied newt (*Cynops orientalis*), and Hong Kong newt (*Paramesotriton hongkongensis*)	Hong Kong SAR, China *Live amphibians exported from Hong Kong to the USA	Cultured/ornamental pet	May and September 2012	Not available	Not available	Presence of ranavirus in 105 of 185 (56.8%) of individual amphibians sampled	PCR	Kolby et al. (2014)
Grouper iridovirus (GIV)	Yellow grouper (*Epinephelus awoara)*	Hsiau Liouchiou Island, near Donggang, Taiwan	Cultured	Not available	Not available	Not available	Not available	Virus isolation, Electron microscopy, Challenge experiments PCR, MCP gene sequencing, and phylogenetic analysis	Murali et al. (2002); Lai et al. (2000)
Ranavirus RCV‐JP (Similar to Rana catesbeiana virus)	North American Bullfrogs (*Lithobates catesbeianus*, formerly *Rana catesbeiana)*	Western Japan	Introduced	September–October 2008	Potentially originated in the USA or by imported infected live freshwater fish from Taiwan	lethargy; palpebral hyperemia, abdominal edema, petechiae, and erythema on the ventral surface, skin ulcers; limb and tail necrosis; and emaciation.	High mortality	Histological examination, electron microscopy, PCR MCP gene sequencing, and phylogenetic analysis	Une, Sakuma, et al. (2009)
*Not specified	Japanese clouded salamander (*Hynobius nebulosus*)	Japan	Captive	Not available	After introduction of some newly collected animals	Skin ulcers	High mortality 100%	PCR, MCP gene sequencing, and phylogenetic analysis	Une, Nakajima, et al. (2009)
*Not specified (no match for registered sequence of any ranavirus)	Poison dart frogs (*Dendrobates* spp. and *Phyllobates* spp.)	Japan	Captive/pets imported	March 2012	After introducing a new imported stock from Netherlands	No skin lesions except adhesion of sloughed skin	Significant morbidity and mortality	PCR, MCP gene sequencing, and phylogenetic analysis	Une et al. (2014)
Inland bearded dragon ranavirus (IBDRV)	Inland bearded dragons (*Pogona vitticeps)*	Saitama Prefecture, Japan	Cultured/pets introduced	December 2014–February 2015	Not available	Skin lesions with recurring multifocal yellowish‐brown crusts	Low mortality	Histopathological examination, PCR, MCP gene sequencing,and phylogenetic analysis	Tamukai et al. (2016)
*Not specified (related to Rana catesbeiana virus JP MCP)	Huanren frog (*Rana huanrenensis*)/tadpoles	Mountain stream in Inje‐gun, Kangwon‐do, South Korea (37° 59′ 1.19′′N 128°29′24.70′′E)	Wild	June 2015	Not available	Slightly swollen skin in tadpoles	High mortality of tadpoles	PCR, MCP sequence sequencing, and phylogenetic analysis	Kwon et al. (2017)
*Not specified (MCP sequence highly resembled Frog virus 3 (FV3)) same haplotype of a previously identified viral sequence collected from Huanren brown frog (*Rana huanrenensis*) from South Korea	Dybowski's brown frog (*Rana dybowskii*)/adults	Moksang‐dong, Gyeyang‐gu, Incheon, South Korea (37° 33′ 34.05″ N, 126° 42′ 12.79″ E)	Wild	March, 2017	Not available	No distinctive external symptoms or erratic behaviors	Not available	PCR, MCP gene sequencing, and phylogenetic analysis	Park et al. (2021)
*Not specified	Boreal digging frog (*Kaloula borealis)*	Eoeun‐dong, Daejeon‐si, South Korea (N36°22'04.25'’, E127°21'35.77'’)	Wild	July, 2016	Not available	Subcutaneous hemorrhage, edema	Low mortality	PCR	Park et al. (2017)
*Not specified	Japanese tree frog (*Hyla japonica*)	Ugok‐ri, Yongju‐myeon, Hapcheon‐gun, South Korea (N35°34'44.82'', E128°04'48.26'')	Wild	June, 2016	Not available	Abdominal edema and hypodermal hemorrhage	Not available	PCR	Park et al. (2017)
*Not specified (MCP gene showed 99% similarity to FV3)	Gold‐spotted Pond Frogs (*Pelophylax chosenicus*, formerly *Rana plancyi chosenica*)	Farm in SinBuk‐myeon, Chuncheon‐si, Gangwon‐do, South Korea	Cultured	2006–2007	Not available	Abscised and incomplete legs in metamorphosing tadpoles, hemorrhage in adults	Not available	PCR, MCP gene Sequencing, and phylogenetic analysis	Kim et al. (2009)
Singapore grouper iridovirus (SGIV)	Grouper (*Epinephelus tauvina)*	Singapore	Cultured	1998	Potentially from the fry which were imported from other SE Asian countries	Hemorrhage and enlargement of spleen	High mortality (more than 90%)	Histopathological examination, PCR, MCP gene sequencing, and phylogenetic analysis	Qin et al. (2003)
Koi ranavirus (KIRV)	Koi (*Cyprinus rubrofuscus)*	India	Cultured/introduced	Not available	Not available	Skin darkening, loss of scales, vertical hanging, uncoordinated swimming, turning upside down, lateral rotation, intermittent surfacing, settling at the bottom laterally	High mortality (often reached 100%)	Virus isolation, transmission electron microscopy, PCR, MCP gene Sequencing. and phylogenetic analysis	George et al. (2015)
Similar damselfish virus (SRDV) (proposed) (MCP gene showed an identity of 99.82% to that of largemouth bass virus)	“Similar Damselfish” (*Pomacentrus similis* Allen, 1991)	India	Cultured/marine ornamental	Not available	Not available	Hemorrhagic lesions, surface ulcerations and damaged caudal fin	High mortality (100% by challenge experiments)	Virus isolation, electron microscopy, challenge experiments, PCR, MCP gene sequencing, and phylogenetic analysis	Sivasankar et al. (2017)
Tiger frog virus (TFV−1998)	Tiger frog (*Hoplobatrachus tigerinus*)	Bangkok, Thailand	Cultured	1998	Not available	Not available	Not available	Electron microscopy, PCR, MCP gene sequencing, and phylogenetic analysis	Sriwanayos et al. (2020)
*Oxyeleotris marmorata* ranavirus (OMRV)	Marbled sleeper goby (*Oxyeleotris marmorata*)	Nakhon Pathom, Thailand	Cultured	2000	Not available	Skin lesions	Not available	Electron microscopy, PCR MCP gene sequencing and phylogenetic analysis	Sriwanayos et al. (2020)
*Poecilia reticulata ranavirus* (PPRV)	Guppy (*Poecilia reticulata*)	Samut Sakhon, Thailand	Cultured/Ornamental Introduced	2001	Not available	Not available	Not available	Electron microscopy, PCR, MCP gene sequencing, and phylogenetic analysis	Sriwanayos et al. (2020)
Goldfish ranavirus (GFRV)	Goldfish (*Carassius auratus*)	Bangkok, Thailand	Cultured/ornamental Introduced	2002	Not available	Not available	Not available	Electron microscopy, PCR, MCP gene sequencing, and phylogenetic analysis	Sriwanayos et al. (2020)
Asian grass frog ranavirus (AGFRV)	Asian grass frog (*Fejervarya limnocharis*)	Sa Kaeo, Thailand	Cultured/imported from Cambodia	2004	Potentially infection originated in Cambodia	Cutaneous ulcerations	Not available	Electron microscopy, PCR, MCP gene sequencing, and phylogenetic analysis	Sriwanayos et al. (2020)
East Asian bullfrog Ranavirus (EABRV)	East Asian bullfrog (*Hoplobatrachusrugulosus)*	Phattalung/ Ratchaburi/Rayong, Thailand	Cultured	2011−2017	Not available	Ulcerative lesions on the dorsal part of the body and legs, cutaneous ulcerations and edema	High mortality	Electron microscopy, PCR, MCP gene sequencing, and phylogenetic analysis	Sriwanayos et al. (2020)
*Not specified Similar to largemouth bass virus (LMBV) and identical to largemouth bass ulcerative syndrome virus (LBUSV)	Barcoo Grunter (*Scortum barcoo)*	Ayutthaya and Phetchaburi Provinces, Thailand	Cultured/introduced from Australia	October to December, 2013	Not available	Extensive hemorrhage and ulceration on skin and muscle	High mortality (Up to 100%)	Challenge assays, PCR, MCP gene sequencing, and phylogenetic analysis	Kayansamruaj et al. (2017)
Grouper iridovirus (GIV)	Tiger grouper hybrid (*Epinephelus sp*.) and Coral trout (*Plectropomus leopardus*)	States of Selangor and Kedah, Peninsular Malaysia	Cultured/introduced mainly from Taiwan and Thailand	2012 to 2014	Not available	Lethargy, darkening of the tail and fins, pale gills, necrosis of fins, sloughing of epidermis, dermal ulceration, enlarged spleen	Not available	Histopathology, PCR, MCP gene sequencing, and phylogenetic analysis	Hazeri et al. (2016), Hazeri et al. (2017)

*Viral name is not specified

It is possible that ranavirus‐caused diseases may have existed undetected in Asia over an extended period, though now coming to be better understood as a result of the widespread application of molecular diagnostic techniques. Alternatively, the detected outbreaks could signal a recent emerging infection spreading rapidly across the world because of the ever‐increasing mobility of pathogens due to global trade of live animals. Application of molecular diagnostic techniques and phylogenomic analyses is important for disease detection and for understanding recombinants that can be more virulent. Phylogenomic analyses using full genomes would explain disease dynamics better. For instance, putative recombinants between FV3, a pathogen widely distributed within wild populations, and CMTV, have caused high pathogenicity. While CMTV‐derived genes associated with virulence are reported in wild strains in Canada, FV3 has been linked to amphibian die‐offs in North America (Vilaca et al., 2019). The latter study provides an insight on how pathogen surveillance and viral dynamics using full genomes can be used to understand the mechanisms of disease origin and spread more clearly. New recombinants arriving through animal trade can pose a high risk to Asia.

It is possible that ranaviruses can be transmitted between ectothermic vertebrate classes through water (Brenes et al., 2014). Further, fish and reptiles might serve as reservoirs for ranavirus, given their ability to live with subclinical infections, which may contribute to the pathogen's persistence, especially when highly susceptible hosts such as amphibians are absent due to seasonal population fluctuations.

Studies from Asia indicate that ranavirus infections cross species barriers, allowing the virus to infect previously uninfected indigenous hosts. Further, given the high mutability of ranaviruses, new strains can emerge (Chen et al., 2013). Some of the introduced host species are known to be infected with ranaviruses in their original locations. One of the best examples is in China, where several imported species that can harbor the infection have been introduced. The Pig frog (*L*. *grylio*) and American Bullfrog (*L*. *catesbeianus*) are popular cultured species introduced from the USA to China. These have been bred and distributed throughout China (Zhang et al., 2001; Zhu & Wang, 2016). Largemouth bass (*M*. *salmoides*), which were identified as being infected with a ranavirus identical to doctor fish virus (DFV) or a strain of DFV, has also been imported to China from the USA (Deng et al., 2011). Largemouth bass virus (LMBV), closely related to DFV, has been recorded in largemouth bass in South Carolina's Santee‐Cooper reservoir, though the origin of the virus has not been confirmed (Mao et al., 1999). Additionally, exotic species being imported as pets, such as red‐eared sliders (*T*. *scripta elegans*) and snapping turtles (*M*. *temminckii*), are known to harbor ranaviruses and are widely traded in China (Moore et al., 2014; Yu et al., 2015).

There are a few records from other countries in the region as well. The barcoo grunter fish (*S*. *barcoo*), which is imported from Australia and cultured in Thailand, was infected with a ranavirus similar to Largemouth bass virus (LMBV) (Kayansamruaj et al., 2017). Inland bearded dragons (*P*. *vitticeps*), a pet species imported to Japan, was found to be infected with Inland bearded dragon ranavirus (IBDRV), though the details of origin are not available (Tamukai et al., 2016). Meanwhile, koi ranavirus (KIRV) has been recorded among imported koi carp (*C*. *rubrofuscus*) in India (George et al., 2015).

In addition, some of the ranavirus infections may already have been transmitted among Asian countries. Asian grass frog ranavirus (AGFRV) has been recorded in Asian grass frogs (*F*. *limnocharis*) imported from Cambodia to Thailand's culture facilities (Sriwanayos et al., 2020). Meanwhile, grouper fry imported from other Southeast Asian countries might have carried the Singapore grouper iridovirus (SGIV) into Singapore and Malaysia (Hazeri et al., 2016, 2017; Qin et al., 2003).

## 10 POTENTIAL ENTRY OF RANAVIRUS TO ASIA THROUGH CULTURED FROGS—AMERICAN BULLFROG (*Lithobates catesbeianus*) AND PIG FROG (*Lithobates grylio*) AS RESERVOIRS

One of the species highlighted so far in this regard is the American bullfrog (*L*. *catesbeianus*), which seems to have played a key role in spreading the pathogen to new locations (Both et al., 2011; Mazzoni et al., 2009; Ruggeri et al., 2019; Schloegel et al., 2009, 2010). Cultured American bullfrogs often carry ranavirus infection FV3 (Miller et al., 2007) and may have served as a vector transmitting the disease to native amphibians and fish in Brazil (Mazzoni et al., 2009; Ruggeri et al., 2019). It is striking those American bullfrogs have been introduced and now occur in nearly 40 countries in Africa, Asia, and North, Central, and South America, and islands of the Mediterranean, South Pacific, and Caribbean (Kraus, 2009). These frogs are of particular concern as vectors of the disease as they are capable of being infected without showing clinical symptoms typical of ranavirosis (Hoverman et al., 2011). Bull frog ranaculture may have facilitated recombination of different species of ranaviruses with enhanced pathogenicity. A chimeric ranavirus that displayed a novel genome arrangement between FV3 and CMTV was observed in a North American farm by Claytor et al. (2017). Further, there is increased risk of new strains, which are different from existing ones, emerging (Oliveira et al., 2020). Thus, the international trade in farmed bullfrogs may have contributed to dispersal of highly pathogenic ranaviruses globally.

There are accounts of North American bullfrogs (*L*. *catesbeianus*) being introduced from Japan (by the Shanghai Fisheries University) to China (Ningbo and Tianjin cities and Guangdong province) between 1958 and 1961 for breeding and distribution (Zhu & Wang, 2016). Viral isolates from southern China were similar to the North American bullfrog isolates from Japan, which indicates the long‐term trade exchange. Interestingly, a ranavirus named RCV‐JP has been identified in cultured North American bullfrogs (*L*. *catesbeianus*) in Japan (Une, Sakuma, et al., 2009). This species is similar to Rana catesbeiana virus identified from North American bullfrogs in a frog farm in the USA (Majji et al., 2006), which suggests that the infection may have persisted in these frogs when they were introduced to Japan from the USA.

Further, *L*. *grylio*, which is native to the south‐eastern USA, has been imported into China for farming purposes. There is a possibility that these imported frogs harbored the virus and were the focus of the initial infection (Zhang et al., 2001). The evidence of recording the Rana grylio virus (RGV), which is similar to the FV3 virus among cultured pig frogs (*L*. *grylio*) in China, could provide more evidence of introduction of the disease from USA, as this species too had been imported.

## 11 POTENTIAL TRANSMISSION TO NATIVE/ENDEMIC SPECIES: JUMPING SPECIES BARRIERS

Several cases indicate that the ranaviral infections in native/endemic host species could potentially jump species barriers of cultured species. Ranavirus infections that have been recorded in Chinese Giant salamander may have been transmitted from pig frogs (*L*. *grylio)* that are routinely fed to farmed Chinese Giant Salamanders (Cunningham et al., 2016). This is supported by the fact that ranavirus from Chinese giant salamanders in Sichuan Province shows a close relationship to ranavirus in pig frogs (Cunningham et al., 2016; Zhou et al., 2013). This hypothesis is further supported by ADRV having been shown to be more closely related to frog (anuran)‐infecting ranaviruses such as CMTV, RGV, FV3, and TFV than ATV, which is the salamander (urodele)‐infecting ranavirus (Chen et al., 2013). The phylogeny we constructed from existing data supports this hypothesis (Figure 2). Further, Triplophysa siluroides ranavirus infecting cultured catfish‐like loach (*Triplophysa siluorides*) in China could potentially have originated in a Chinese giant salamander farm, in which water may have been the vector for the CGSV infection (the salamander farm is located approximately 1 km upstream of the loach farm; Deng et al., 2020).

Potential interclass transmission of TFVs in Thailand may have occurred in live animal markets, where tadpoles and frogs are housed in open containers next to ornamental fishes, in addition to the feeding of juvenile frogs to large predatory ornamental fishes (Sriwanayos et al., 2020).

## 12 BIOSECURITY AND PREVENTING FURTHER ENTRY OF RANAVIRUSES TO ASIA

Import risk analysis (IRA) is a procedure used to determine the threat of a pathogen entering a system with international trade in animals and their products. This has been largely driven by the sanitary and phytosanitary (SPS) agreement of the World Trade Organization (WTO) and the IRA standard established by the World Organization for Animal Health (OIE), which provides an IRA standard aquatic and terrestrial animal health codes (Peeler et al., 2013; World Organization for Animal Health (OIE), 2019, 2019). The principal aim of import risk analysis is to provide importing countries with an objective and defensible method of assessing the disease risks associated with the importation of animals and their products.

IRAs can also be used to establish or revise trade or translocation guidelines for wildlife that could be subclinically infected with a pathogen (Smith et al., 2009). Risk analysis on wildlife species in trade, pre‐border pathogen screening, and voluntary support should help reduce costs associated with species invasion, as well as protecting the public and enhancing environmental and animal health (Smith et al., 2009). Harmonia+, a framework based on 30 questions, can be used to distinguish the components of invasion (D’hondt et al., 2015). Pandora+ is a protocol that assesses the risk of a particular pathogen or parasite being introduced by a particular host species (D’hondt et al., 2015). It has the same structure as Harmonia+ and consists of 13 key questions. Pandora+, along with Harmonia+, can successfully be used to assess the risk of pathogens in invasive species. The Pandora+ protocol has been successfully used to demonstrate a high risk for pathogens with potential to affect amphibians from neighboring regions in Sinaloa, Mexico, following the first report of a ranavirus outbreak in farmed American bullfrogs (*L*. *catesbeianus*; Saucedo et al., 2019). These techniques can successfully be used also to assess risk in Asia and prevent further introductions of high‐risk host species to the region. Further, it is important to carry out disease surveillance for all ectothermic species now being cultured to prevent further disease spread to wild species.

When a ranavirus case is reported, it is important to report it to responsible government agencies and to the World Organization for Animal Health (OIE) through relevant channels. There is an urgent need for improved biosecurity practices and a better understanding of the pathogen–host–environment imbalance often created under artificial culture conditions (Chinchar & Waltzek, 2014) in both aquaculture/ranaculture and the pet trade in Asia, especially given that the existing measures are clearly inadequate. Disease prevention, driven by legislation and effective regulation, is fundamental to the sustainability of the aquaculture industry (Gudding, 2012). Meanwhile, existing national, regional, and international laws and regulations can be successfully used to improve biosecurity measures related to aquaculture practices for minimizing the risk of disease emergence and transmission.

Identification of national priorities for aquatic animal health management and the development of national strategies are important to all the countries of Asia‐Pacific (Aus Aid & Network of Aquaculture Centres, 2006). Since ranaviruses pose a high risk to Asia, each country in the region should be equipped to conduct import risk analysis. The authorities dealing with animal health should screen imported consignments of live ectothermic animals and their products for pathogens according to established risk assessment criteria before allowing them in. The records of the presence of ranaviruses should be shared transparently in the region as collective effort is important in controlling the disease. Developing such a framework for surveillance and reporting, as well as a framework for contingency planning in Asia, are vital steps in controlling infections in the region. Many countries, meanwhile, lack infrastructure and expertise when it comes to disease diagnosis, surveillance, quarantine, and risk analysis needed to control infections in the region. Therefore, an effective legal framework, along with enhanced disease surveillance and biosecurity measures, is required to control potential pathogen introductions, as well as to minimize risk of disease transmission.

## 13 CONCLUSIONS AND FUTURE DIRECTIONS

Ranaviral infections have up to now been recorded in at least nine countries and/or administrative regions of Asia. Our review highlights that the surveillance effort in Asia is inadequate. China has lead surveillance and research in the region. However, most of the surveillance work carried out in the rest of Asia has been sporadic and opportunistic, and confined to species of economic value given that investigations have focused on major outbreaks and die‐offs. Some countries with high potential host diversity, such as Laos, Myanmar, Brunei, Bangladesh, Bhutan, Nepal, Pakistan, and Sri Lanka, lack any records of surveillance work thus far. There is an urgent need to establish the possible presence of ranaviruses in these countries. Infections have been recorded in all the three ectothermic vertebrate classes both in cultured species (indigenous/endemic or introduced) and in wild species (Table 1 and Figure 2). Infections spread rapidly in cultured populations with low genetic variability. A large number of animals belonging to different classes and species are stocked in small spaces, causing stress in animals, while feeding diseased animals to other species can facilitate spread of infection. Introduction of American bullfrogs (*L*. *catesbeianus*) and Pig frogs (*L*. *grylio*) for ranaculture, largemouth bass (*M*. *salmoides*) for aquaculture, and pet species such as Red‐eared sliders (*T*. *scripta elegans*), which are known to carry ranaviral infections in their original home ranges, appears to exacerbate the dispersal of infection in Asia. The growing popular industry of Grouper culture could potentially spread Grouper iridovirus (GIV) in the whole region. Further, infected animals may be deliberately or accidently released to the wild, while infection could also be transmitted via other media such as contaminated waste. Given that ranaviruses are often reported during summer months, rising temperatures associated with climate change may facilitate ranavirus spread. Mass die‐offs have been recorded in several countries such as China and Japan. So far, no effective treatment to reduce mortality or morbidity has been found. Therefore, control measures, such as limiting international trade of animals and screening for disease, must be strictly followed. Molecular diagnostic techniques can be successfully used to observe the phylogenetic relationships and their host ranges, to detect the possible original sources of introduction. Well‐planned, widely distributed systematic screening is essential to understand the prevalence and impact in Asia, to conserve the biodiversity and to safeguard the widely established and economically important aquaculture industry.

## CONFLICT OF INTEREST

None to declare.

## AUTHOR CONTRIBUTIONS

**Jayampathi Herath:** Conceptualization (equal); Formal analysis (lead); Investigation (lead); Methodology (lead); Project administration (equal); Validation (equal); Visualization (equal); Writing‐original draft (equal); Writing‐review & editing (equal). **Gajaba Ellepola:** Conceptualization (equal); Formal analysis (equal); Investigation (equal); Methodology (equal); Visualization (equal); Writing‐original draft (supporting); Writing‐review & editing (equal). **Madhava Meegaskumbura:** Conceptualization (equal); Investigation (equal); Project administration (equal); Resources (lead); Supervision (lead); Writing‐original draft (equal); Writing‐review & editing (equal).

## Data Availability

Data sharing: not applicable. All data and sources used in the study are provided within the framework of the study.
